# Surgical and medical second trimester abortion in South Africa: A cross-sectional study

**DOI:** 10.1186/1472-6963-11-224

**Published:** 2011-09-19

**Authors:** Daniel Grossman, Deborah Constant, Naomi Lince, Marijke Alblas, Kelly Blanchard, Jane Harries

**Affiliations:** 1 Ibis Reproductive Health, Oakland, CA, 94612, USA; 2 Bixby Center for Global Reproductive Health and San Francisco General Hospital, Department of Obstetrics, Gynecology and Reproductive Sciences, University of California, San Francisco, CA, 94143-0744, USA; 3 Women's Health Research Unit, School of Public Health and Family Medicine, University of Cape Town, Cape Town, 7925, South Africa; 4 Ibis Reproductive Health, Johannesburg, 2041, South Africa; 5 Independent consultant, Cape Town, 7925, South Africa; 6 Ibis Reproductive Health, Cambridge, MA, 02238, USA

## Abstract

**Background:**

A high percentage of abortions performed in South Africa are in the second trimester. However, little research focuses on women's experiences seeking second trimester abortion or the efficacy and safety of these services.

The objectives are to document clinical and acceptability outcomes of second trimester medical and surgical abortion as performed at public hospitals in the Western Cape Province.

**Methods:**

We performed a cross-sectional study of women undergoing abortion at 12.1-20.9 weeks at five hospitals in Western Cape Province, South Africa in 2008. Two hundred and twenty women underwent D&E with misoprostol cervical priming, and 84 underwent induction with misoprostol alone. Information was obtained about the procedure and immediate complications, and women were interviewed after recovery.

**Results:**

Median gestational age at abortion was earlier for D&E clients compared to induction (16.0 weeks vs. 18.1 weeks, p < 0.001). D&E clients reported shorter intervals between first clinic visit and abortion (median 17 vs. 30 days, p < 0.001). D&E was more effective than induction (99.5% vs. 50.0% of cases completed on-site without unplanned surgical procedure, p < 0.001). Although immediate complications were similar (43.8% D&E vs. 52.4% induction), all three major complications occurred with induction. Early fetal expulsion occurred in 43.3% of D&E cases. While D&E clients reported higher pain levels and emotional discomfort, most women were satisfied with their experience.

**Conclusions:**

As currently performed in South Africa, second trimester abortions by D&E were more effective than induction procedures, required shorter hospital stay, had fewer major immediate complications and were associated with shorter delays accessing care. Both services can be improved by implementing evidence-based protocols.

## Background

Both medical and surgical abortion procedures are used in the second trimester. Intra-amniotic instillation procedures are no longer recommended because they are associated with more complications than other procedures [1]. Modern medical methods include induction with mifepristone and misoprostol or with misoprostol alone. The combined mifepristone regimen is significantly more effective and results in a shorter induction time than misoprostol used alone [2]. In South Africa, misoprostol alone is currently the standard of care for medical termination of pregnancy in the second trimester within the public health sector. Dilation and evacuation (D&E) is the surgical method recommended by the World Health Organization [3].

The few studies that have compared medical and surgical second trimester abortion techniques all have been conducted in developed country contexts [4-8]. One small randomized controlled trial (RCT) comparing D&E to the mifepristone regimen at 14-19 weeks' gestation in the United States (U.S.) found that women undergoing medical induction had a higher risk of complications (RR 6.0, 95% CI 0.9-40.3) [6]. Another RCT in the United Kingdom found that complications were similar between the two methods, although women undergoing medical induction reported more pain and found the procedure less acceptable compared to those undergoing D&E [5]. A retrospective audit of second-trimester procedures at one facility in New Zealand found that evacuation for retained products of conception was significantly more common among women undergoing medical induction [4]. Another retrospective cohort study from the U.S. of women undergoing D&E or medical induction, mostly with misoprostol used alone, found that adverse events were significantly more common among medical induction patients (29% vs. 4%, p < 0.001) [7]. In particular, failure of initial procedure and retained tissue requiring curettage were more common with medical induction. In a retrospective cohort study of U.S. women seeking termination for fetal anomalies, the adjusted risk ratio of having any complication was significantly higher for women undergoing induction with misoprostol compared to D&E (RR 8.5, 95% CI 3.7-19.8) [8]. A recent review of large case series of second trimester abortion procedures also found that medical induction with the mifepristone regimen was associated with a higher proportion of incomplete abortions, as well as a possible higher risk of hemorrhage requiring transfusion and mild infection, compared with D&E [9].

Information about the safety and efficacy of these termination methods is particularly relevant in countries that are beginning to scale up second trimester services. The Choice on Termination of Pregnancy Act was passed in 1996 in South Africa, allowing abortion up to 12 weeks' gestation on request; termination up to 20 weeks is legal for several indications, including socio-economic hardship [10]. Approximately 25% of abortions in South Africa are performed after 12 weeks [11], whereas in most developed countries approximately 10% of abortions are in the second trimester [12,13]. Most second trimester abortions in South Africa are performed by medical induction with misoprostol alone, although in Western Cape Province, a roving team of three doctors provides D&E in the public sector [14] Due to the lack of trained D&E providers in other areas of the country, hysterotomy is sometimes performed in cases of failed induction [15].

The objective of this study was to describe women's experiences seeking and undergoing second trimester abortion at public sector hospitals in Western Cape Province, including documenting the efficacy, safety and acceptability of medical and surgical abortion as currently performed.

## Methods

We performed a cross-sectional study of women undergoing either D&E or second trimester medical induction with misoprostol alone. We investigated access to and acceptability of D&E and medical induction by interviewing women after completion of their abortion. Length of procedure, medications used, safety and efficacy were compared using clinical information collected during or after the procedure.

The study was approved by the University of Cape Town Research Ethics Committee, Stellenbosch University Committee for Human Research and Allendale Investigational Review Board. Permission to perform the research was obtained from the Provincial Department of Health, Western Cape. All participants provided written informed consent, and confidentiality and anonymity were ensured. Study procedures were carried out in accordance with the revised version of the Helsinki Declaration and with the standards of Good Clinical Practice.

The study was conducted between February and July 2008 at five public sector hospitals in Western Cape Province, South Africa. These sites were five of the nine public hospitals in the province that provided second trimester abortion services, either by D&E or medical induction, during the study period. The remaining four hospitals performing second trimester abortion were not included in the study due to logistical reasons which included distance, low volume and permission being denied at one facility. Women generally were referred to these hospitals based on regional proximity, and clients were not offered a choice of which facility to attend. No hospital offered both D&E and medical induction, and therefore women did not have a choice of abortion method after presenting to the facility.

Three of the study hospitals offered outpatient D&E services, and the other two offered medical induction on an inpatient basis. Of the five sites, two D&E sites and one induction site served a primarily urban population, while the other sites served a more peri-urban and rural population. We collected data at each site on the total number of second trimester abortions performed during the study period by reviewing logs of abortion procedures maintained at the facilities.

All women attending these hospitals for second trimester abortion on days when study interviewers were present were invited to participate in the study. Eligibility was assessed prior to the abortion, and criteria included age 18 years or older, gestational age between 12.1 weeks and 20.9 weeks, and ability to communicate in English or Xhosa. All participants provided written informed consent before enrollment to both an interview and hospital record review.

Bilingual interviewers administered structured questionnaires in a private location at the hospital after the woman recovered from the abortion. Data were obtained on age, spoken language, education, employment, housing conditions and reproductive history. Information was also obtained about the number and timing of clinic visits leading up to admission for the current abortion. After initial data collection, the long delay between date of the first clinic visit and date of admission for the abortion as reported by the participant became apparent. To substantiate this finding, additional information was collected on the date and result of the ultrasound scan from participants' charts. Although the ultrasound may not have been performed at the first clinic visit, it was the only documented record of participants' access to abortion-related care.

For details relating to the abortion experience itself, clients were asked to rate the following on a 5-point Likert scale: overall satisfaction with the abortion, pain and emotional discomfort.

For participants undergoing medical induction, a procedure form was completed by the attending doctor or by the study investigators following discharge of the participant. Data were collected on gestational age by ultrasound, date of initial ultrasound, duration of procedure (from first misoprostol dose to placental expulsion or surgical procedure, if performed), duration of hospital stay, timing and dosage of misoprostol and any other medications, and details of additional surgery, if performed. When forms were incomplete, participants' medical records were reviewed. For participants undergoing D&E, the procedure form was completed by the doctor performing the abortion immediately after the procedure. Gestational age by ultrasound, duration of surgical procedure, details of the procedure, and whether fetal expulsion occurred prior to surgery were noted. Because most of the relevant information on the D&E procedure was not documented in the medical record, it was not possible to collect this information later if the form was incomplete.

The primary outcome of the study was procedure efficacy, defined as complete abortion performed on site, with no need for repeat surgical procedure in the case of D&E, or no need for any surgical intervention in the case of induction. Secondary outcomes included safety and acceptability. Safety was assessed by measuring the prevalence of immediate major complications (death, seizure, injury to organ requiring abdominal surgery, or hemorrhage requiring transfusion) or minor complications (hemorrhage without transfusion, fever, cervico-vaginal trauma, transfer to another facility because of inability to complete procedure, unplanned surgical procedure (for induction) and fetal expulsion prior to procedure (for D&E)) [16]. Because it was not standard at these sites to have women return for a follow-up visit, information on delayed complications was not available. Acceptability was determined by overall satisfaction, pain and emotional discomfort, and likelihood of recommending the abortion method to others.

Data were analyzed using Stata (version 10, College Station, TX). We summarized data on participant characteristics, abortion-related hospital and clinic visits, procedure details, efficacy, complications and acceptability by abortion method using proportions for categorical variables and medians for continuous variables. 95% confidence intervals (CI) were calculated for proportions, and proportions were compared using chi-square tests or Fisher's exact tests when appropriate. Where medians were compared, Wilcoxon rank sum tests were used. Two-sided significance tests were used throughout. Missing values were excluded from the analysis, and valid percentages are reported for all results. Statistical comparisons were performed by procedure rather than by site as numbers were often too small to allow for analysis by site.

The number of subjects recruited was sufficient to detect a difference in efficacy between 85% for induction and 98% for D&E, with 90% power and two-sided alpha of 0.05.

## Results

**Figure 1:**
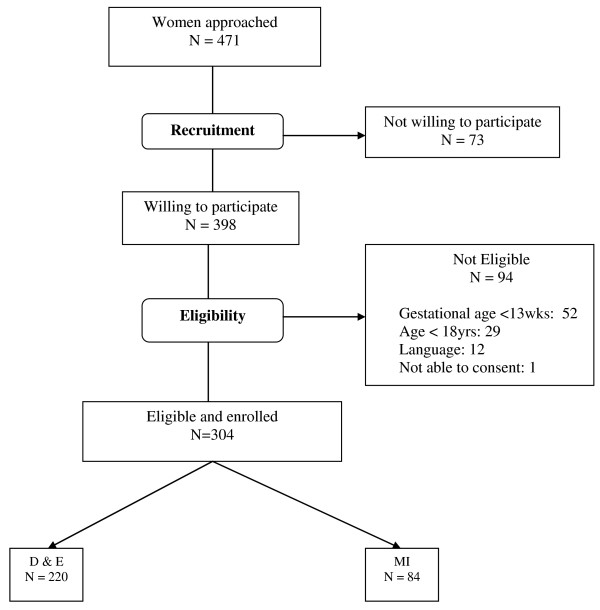
**Flowchart of participant enrollment**.

During the data collection period, 746 D&E and 168 second trimester medical inductions were performed at the study sites. Two hundred twenty women undergoing D&E (29.5% of all D&E cases) and 84 women undergoing medical induction (50% of all induction cases) were enrolled in the study. Figure 1 demonstrates the recruitment process, including the reasons why women were not eligible. Participant characteristics, shown in Table 1, were similar in both groups except for gestational age on admission (date of procedure for D&E and date of hospital admission for medical induction) and the proportion speaking Afrikaans. Median gestational age for D&E clients was 15.0 weeks (inter-quartile range (IQR) 14.0 - 16.6 weeks) and for induction clients was 18.1 weeks (IQR 17.6 - 18.9 weeks). For all participants, median age was 25 years; 51% had completed secondary school, and just 6.5% had completed only grade 8 or lower. Most women (80%) had one or more children, and most (71%) had had a vaginal delivery, while 9% had previously undergone a caesarian section. Few participants (4%) reported having had a prior abortion. Sixty-five percent were not paid for work, 65% lived in formal housing (house or flat), as opposed to informal housing (shack), and for most participants (79%) there were two or fewer people per room in the place where they lived. Xhosa was the most common language spoken at home (69%), followed by English (26%) and Afrikaans (22%). More D&E clients spoke Afrikaans at home, which was likely related to the catchment areas of the hospitals.

**Table 1:** Participant characteristics

Characteristic	**D&E**^1^ (n = 220)	Medical induction (n = 84)	P value
Age (years)			0.523

18-25	112 (51%)	47 (56%)	

26-35	89 (41%)	33 (39%)	

>35	17 (8%)	5 (5%)	

Education (Grade)			0.765

< Gr 12	107 (49%)	35 (47%)	

Gr 12	110 (51%)	39 (53%)	

Parity			

0	49 (23%)	15 (18%)	0.167

1	78 (36%)	40 (48%)	

> = 2	91 (42%)	29 (35%)	

Gestational age on admission (weeks)			**<0.001**

12.1-16.0	144 (66%)	12 (14%)	

16.1-18.0	47 (22%)	27 (32%)	

18.1-20.9	28 (13%)	45 (54%)	

Prior vaginal delivery	149 (68%)	58 (78%)	0.101

Prior Caesarean Section	21 (10%)	5 (7%)	0.453

Prior Abortion	6 (3%)	5 (7%)	0.118

Housing			0.801

Formal (House or Flat)	142 (65%)	47 (64%)	

Informal (Shack)	76 (35%)	27 (37%)	

Persons per room			0.550

< = 2	175 (80%)	57 (68%)	

>2	43 (20%)	17 (23%)	

Languages spoken			

Xhosa	148 (68%)	52 (70%)	0.703

English	57 (26%)	18 (24%)	0.757

Afrikaans	60 (28%)	5 (7%)	**<0.001**

Paid for Work	74 (34%)	28 (38%)	0.596

^1 ^D&E Dilation and evacuation

Information about the number and timing of visits prior to the abortion is shown in Table 2. The majority of participants reported three or more clinic or hospital visits related to the abortion, and induction clients reported significantly more visits (p < 0.001). Substantial delays occurred between the date of the first clinic visit reported by the participant and the date of admission for the abortion. The median delay was significantly longer for induction clients (30 days, IQR 21-56 days) than for D&E clients (17 days, IQR 8-30 days, p < 0.001). Based on the date of the reported visit, 40% of D&E clients and 39% of induction clients were at 12 weeks' gestation or earlier at the time of the first clinic visit. Median gestational age at ultrasound was slightly earlier for induction clients, and the time interval from the date of the ultrasound to the date of admission was significantly longer for induction clients (31 days, IQR 12-41) than for D&E clients (6 days, IQR 2-9, p < 0.001).

**Table 2:** Number and timing of visits prior to abortion

	D&E (n = 220)	Medical induction (n = 84)	P value
Total number of clinic or hospital visits related to abortion (N, %)			**<0.001**

1	0 (0%)	1 (1.4%)	

2	54 (25.1%)	8 (10.8%)	

3	117 (54.4%)	17 (23.0%)	

4 or more	44 (20.5%)	48 (64.9%)	

Number of days between first clinic or hospital visit and abortion admission (Median, IQR)^2^	17 (8 - 30)	30 (21-56)	**<0.001**

Gestational age in weeks at time of ultrasound (weeks)^a ^(Median, IQR)	14.0 (13.0 - 15.4)	13.9 (11.9 - 15.4)	**0.045**

< = 12 weeks at time of ultrasound (N, %)	13 (8.1%)	24 (30.0%)	**<0.001**

Number of days between ultrasound and abortion admission (Median, IQR)^a^	6 (2 - 9)	31 (12 - 41)	**<0.001**

^2 ^Inter-quartile Range

^a ^Data available for 161 D&E clients and 80 medical induction clients

More induction clients than D&E clients were in the first trimester at the time of their ultrasound (30.0% vs. 8.1%, p < 0.001). These data were available for 161 D&E clients and 80 induction clients.

Table 3 shows information about the abortion procedure, including complications, which was available for 203 D&E clients and 84 induction clients. The median duration of the surgical procedure for D&E was 10 minutes (IQR 5 - 10 minutes), and median time from first misoprostol dose to completion of D&E was 13.3 hours (IQR 12.1 - 15.1 hours), with no overnight stays in hospital. The median duration from first misoprostol dose to completion of the induction procedure was 23 hours (IQR 17 - 30 hours), and all but one woman required an overnight stay in hospital. The median duration of hospitalization for induction clients was 1.3 days (IQR 1.1-2.1 days), with a maximum hospital stay of 11.1 days.

**Table 3:** Procedure details, efficacy and immediate complications

	D&E (n = 203)	Medical induction (n = 84)	P value
Length of surgical procedure (Median, IQR)	10 minutes (5-10 minutes)	N/A^3^	

Time from 1st dose misoprostol to completion of TOP (Median, IQR)	13.3 hrs (12.1-15.1)	23 hours (17-30)	**<0.001**

Number of misoprostol doses (Median, IQR)	3 (3-3)	5 (3-5)	**<0.001**

			

Medications received			

Antibiotics	14 (7%)	65 (77%)	**<0.001**

Analgesics/anxiolytics	38 (19%)	18 (21%)	0.585

Antiemetics	0 (0%)	7 (8%)	**<0.001**

			

Efficacy^a^	202 (99.5%) (95% CI: 97.29% - 99.98%)	42 (50%) (95% CI: 39.4% - 60.6%)	**<0.001**

			

Major Complications			

Hemorrhage requiring transfusion	0 (0.0%)	3 (2.4%)	**0.024**

			

Minor Complications			

Hemorrhage not requiring transfusion	0 (0%)	1 (1%)	0.293

Fever >38 degrees C	0 (0%)	1 (1%)	0.293

Additional curettage performed	0 (0%)	40 (48%)	**< 0.001**

Incomplete abortion, absconded prior to treatment	0 (0%)	1 (1%)	0.293

Transfer to another facility to complete abortion	1 (1%)	1 (1%)	0.500

Expulsion of fetus prior to D&E	87 (43%)	N/A	N/A

Any minor complication	88 (43%)	42 (50%)	0.362

Any major or minor complication	88 (43%)	44 (52%)	0.193

^3 ^Not applicable

^a ^Efficacy was defined as complete abortion performed on-site with no need for repeat surgical procedure in the case of D&E, or no need for any surgical intervention in the case of medical induction

Misoprostol was used to prepare the cervix prior to D&E at the sites offering this method, although the regimen varied both between and within sites. An initial dose of misoprostol 400 mcg was taken sublingually at home either the night before or the morning of the abortion procedure. Thereafter the dose was repeated at four-hourly intervals, usually up to a total of three doses. However, at one D&E site, only two misoprostol doses were taken prior to admission. When deemed necessary by the doctor or nurse, additional doses were given, up to a maximum of eight. D&E was performed using paracervical block with 20 cc of 1% lidocaine.

The medical induction sites also followed somewhat different regimens of misoprostol. The initial misoprostol dose was either 600 mcg administered vaginally or 400 mcg taken orally. Thereafter, misoprostol 400 mcg was administered orally every four hours. The median number of misoprostol doses administered was 5 (IQR 3-5), however the maximum number of doses was as high as 36. When indicated, sharp or suction curettage was performed.

Although frequently provided to induction clients (77.4%) prophylactic antibiotics were rarely given to D&E clients (6.9%). Only 18.6% of D&E clients and 21.4% of induction clients received either analgesics or anxiolytics. Antiemetics were provided to a small number of induction clients (8.3%).

D&E was significantly more effective than induction; only one D&E client with clinical data required transfer to another facility because of inadequate cervical dilation (efficacy 99.5%, 95% CI 97.3-99.98%). Among 84 induction clients, 40 required curettage for incomplete abortion, one was diagnosed with incomplete abortion but left the hospital before having the procedure, and one was transferred to another facility because of a failed induction (efficacy 50.0%, 95% CI 39.4-60.6%). Of the 40 cases of incomplete abortion, 36 (90%) were treated with sharp curettage, 2 (5%) with suction curettage and 2 (5%) with other procedures. It is important to note, however, that we were not able to collect information about incomplete abortion after either procedure that might have required delayed re-aspiration after hospital discharge.

As shown in Table 3, the only major immediate complications were three cases of hemorrhage requiring transfusion. Major complications were significantly more prevalent after induction compared to D&E, as they all occurred among induction clients (p = 0.024). The prevalence of minor immediate complications, however, was similar between the procedures. Fetal expulsion prior to surgery was observed among 42.9% of D&E clients and was especially frequent at the sites that initiated misoprostol for cervical priming the night prior to the abortion. In addition to the cases of failed and incomplete abortion mentioned above, one induction client had hemorrhage that did not require transfusion, and one had a fever.

Of the 80 D&E clients who expelled the fetus prior to the procedure and answered the question, 42 (52.5%) expelled it at home, 4 (5%) during transport, and the remainder (42.5%) expelled at the hospital. Upon noticing the fetal expulsion, women reported feeling scared (32.5%), surprised (30%), sad (13.8%) or other emotions; 16.3% expressed relief that the abortion was successful and complete.

Information on women's symptoms and acceptability of the procedures is shown in Table 4. Women undergoing D&E reported significantly higher pain levels than those undergoing induction (p = 0.001). The prevalence of other symptoms was similar for both groups. Significantly more D&E clients reported high levels of emotional discomfort associated with the abortion compared with induction clients, although most clients (> 84%) were satisfied with the emotional support received during the procedure. Overall, there were very high levels of satisfaction with both abortion procedures, and the majority of women (73%) said that they would recommend their method to a friend who needed a second trimester abortion.

**Table 4:** Symptoms and acceptability of abortion experience

	D&E (n = 220)	Medical induction (n = 84)	P value
Overall pain during entire abortion experience			**0.001**

Extreme pain	75 (34%)	23 (31%)	

High pain	50 (23%)	10 (14%)	

Moderate pain	40 (18%)	11 (15%)	

Slight pain	38 (17%)	11 (15%)	

No pain	15 (7%)	19 (26%)	

			

Symptoms reported			

Nausea	15 (7%)	2 (3%)	0.255

Vomiting	57 (26%)	23 (31%)	0.452

Dizziness	63 (29%)	25 (34%)	0.465

Diarrhea	83 (38%)	40 (54%)	**0.020**

Tiredness	107 (49%)	40 (54%)	0.575

Pain in lower abdomen	214 (98%)	74 (100%)	0.573

Breast tenderness	106 (49%)	25 (34%)	**0.031**

Headache	84 (39%)	27 (37%)	0.783

Cold	89 (41%)	29 (39%)	0.891

Sweating/hot/fever	13 (6%)	3 (4%)	0.769

Other symptoms	31 (14%)	2 (3%)	**0.005**

			

Overall emotional discomfort with abortion			**0.017**

Moderate, high or extreme discomfort	108 (50%)	25 (34%)	

None or slight discomfort	109 (50%)	49 (66%)	

			

Overall satisfaction with abortion			1.000

Very or somewhat satisfied	207 (95%)	71 (96%)	

Neutral or somewhat or very dissatisfied	11 (5%)	3 (4%)	

			

Would recommend the abortion method to a friend who needed one at same gestational age			0.995

Highly or somewhat agree	159 (73%)	54 (73.0%)	

Neutral or somewhat or highly disagree	59 (27%)	20 (27%)	

## Discussion

This is the first comparative study documenting D&E and medical induction services in a developing country setting, and the first in South Africa, where second trimester abortions are more prevalent than other countries. Despite the different context, our findings on the safety and efficacy of D&E and second trimester medical induction are similar to those reported in more developed country settings [4-8,17]. Both D&E and medical induction are being provided safely in this context, although induction frequently required curettage to complete the procedure and had a higher proportion of major immediate complications. The association of induction with hemorrhage requiring transfusion is concerning and merits further research; a similar trend was observed in a review of case series [9], while a large registry study from Finland found no increased risk of hemorrhage with second trimester medical induction compared to first trimester medical abortion [17].

Compared to the D&E services, the medical induction services had limited capacity, as demonstrated by the smaller number of induction cases performed during the study period. The limited capacity of this service likely contributed to the delays between presentation and abortion that we documented among induction clients, although there may have been other reasons as well. Prior research in South Africa found that women seeking second trimester abortion reported structural barriers within the public health system as factors that contributed to presenting later in pregnancy [18]. Every additional week of gestation confers a significant increase in the risk of mortality with abortion [19], and it is critical that health systems work to improve the referral process to minimize these delays. It is particularly concerning that almost one third of induction clients were in the first trimester at the time of their ultrasound, when they would have been eligible for a manual vacuum aspiration (MVA) procedure performed by a midwife or nurse.

Taken together, these findings suggest that D&E might be the superior method to provide second trimester abortion in South Africa and similar settings. D&E using MVA has been introduced successfully in Vietnam [20] and Nepal [21]. However, there is a critical shortage of trained D&E providers in South Africa, and doctors are reluctant to be involved in D&E services [14,22]. Investments in training, including pre-service training in medical school, as well as values clarification exercises with physicians [23] are needed to create a cadre of skilled D&E providers in this country.

Our results highlight several areas for improvement in the second trimester abortion services in the Western Cape. We found that 43% of D&E clients expelled the fetus prior to the procedure, which was frequently traumatic for women, and this was likely related to the repeated doses of misoprostol given as much as 12 hours before the procedure. Indeed, for the women who experienced fetal expulsion, their abortion procedure was more similar to medical induction than D&E. Although there is limited published evidence on the use of misoprostol instead of osmotic dilators for cervical preparation before D&E, a single dose of 400 mcg is the most commonly studied regimen [24]. More research is needed on cervical priming protocols appropriate for settings where laminaria are not easily accessible, as is the case in South Africa. The prolonged cramping associated with misoprostol prior to D&E might also account for the higher pain levels reported by women undergoing this procedure compared to induction, as well as the fact that few D&E clients received analgesia. In contrast, both RCTs found that induction clients reported significantly more pain than D&E clients, although women in the latter group received general anesthesia in both studies [5,6]. It is concerning that analgesia use was low at all but one of the study sites. In addition to standardizing protocols for pain management with second trimester abortion, research is needed to understand clinicians' reluctance to provide analgesia, which may be related to punitive attitudes toward abortion.

The medical induction protocol used in study sites had low efficacy compared to published studies using repeated vaginal or sublingual doses of misoprostol [25]. In addition, using mifepristone together with misoprostol has been demonstrated to significantly improve efficacy and reduce the induction time [2]. Mifepristone is not registered in South Africa for use in the second trimester and is therefore not available in public hospitals for this indication. We also found that sharp curettage was used almost exclusively in cases of incomplete abortion, despite the evidence supporting the superiority of suction curettage for this indication [3].

This study has several limitations. The observational nature of the study may have biased our findings, although it is important to stress that women's allocation to treatment was related to place of residence and hospital catchment area, rather than personal choice. Still, unmeasured differences between the populations undergoing the two procedures might have accounted for some of the observed differences. Comparison between the two abortion methods is also made difficult by the fact that a standardized protocol was not used in all sites. A randomized controlled trial of D&E and medical induction of sufficient size is needed to better compare these methods, although both published RCTs have found that recruitment for such a study is difficult [5,6]. Another limitation relates to our lack of follow-up and inability to capture delayed complications. This is especially true of women undergoing D&E, since they were observed for a relatively short period of time. More research is needed on the long-term complications of both of these methods. Due to the high volume of procedures performed on days that the D&E service operated, we were only able to enroll a smaller percent of all D&E cases (29.5%) compared to all medical induction cases (50%). However, the D&E sample is representative of all three D&E providers in the Western Cape and is sufficiently large to be representative of the D&E service overall. Finally, while our study sites are likely representative of the Western Cape, we cannot generalize beyond this province to other areas of South Africa or other countries.

## Conclusion

In the decade since legalizing abortion, South Africa has made important advances in reducing abortion-related morbidity and mortality [26,27]. This study shows that currently in the Western Cape, second trimester abortions by D&E were more effective than medical procedures, required shorter hospital stay, had fewer major immediate complications and were associated with shorter delays accessing care. The delays for women undergoing medical induction were so long that they appeared to push some women into the second trimester even though they initially presented during the first trimester.

Second trimester abortion services are particularly challenging due to the training investments that are required. While clinical trial data from more developed countries can be useful to guide the implementation of services, it is critical that information about local capacity and resources also be considered. Because access to good quality second trimester services is an essential part of safe abortion services [28], implementing the service changes described here could serve as a model for other countries that are beginning to offer later abortion care. In addition, more efforts are needed to expand access to contraception, pregnancy testing and first trimester abortion, including medication abortion, in order to reduce the proportion of abortions performed in the second trimester in South Africa.

## Competing interests

The authors declare that they have no competing interests.

## Authors' contributions

DG, DC, NL, and JH designed the study, supervised data collection and analysis and interpreted the results. MA and KB contributed to the study design and interpretation of results. DG and DC drafted the article and others commented. All authors have approved the final version.

## Pre-publication history

The pre-publication history for this paper can be accessed here:

http://www.biomedcentral.com/1472-6963/11/224/prepub
